# Duplicated Flagellins in Pseudomonas Divergently Contribute to Motility and Plant Immune Elicitation

**DOI:** 10.1128/spectrum.03621-22

**Published:** 2023-01-11

**Authors:** Yuan Luo, Jing Wang, Yi-Lin Gu, Li-Qun Zhang, Hai-Lei Wei

**Affiliations:** a Key Laboratory of Microbial Resources Collection and Preservation, Ministry of Agriculture and Rural Affairs, Institute of Agricultural Resources and Regional Planning, Chinese Academy of Agricultural Sciences, Beijing, China; b Department of Plant Pathology, China Agricultural University, Beijing, China; USDA – San Joaquin Valley Agricultural Sciences Center

**Keywords:** *Pseudomonas*, flagellin, Flg22, motility, plant immunity, flagella, plant-microbe interactions

## Abstract

Flagellins are the main constituents of the flagellar filaments that provide bacterial motility, chemotactic ability, and host immune elicitation ability. Although the functions of flagellins have been extensively studied in bacteria with a single flagellin-encoding gene, the function of multiple flagellin-encoding genes in a single bacterial species is largely unknown. Here, the model plant-growth-promoting bacterium Pseudomonas kilonensis F113 was used to decipher the divergent functions of duplicated flagellins. We demonstrate that the two flagellins (FliC-1 and FliC-2) in 12 Pseudomonas strains, including F113, are evolutionarily distinct. Only the *fliC-1* gene but not the *fliC-2* gene in strain F113 is responsible for flagellar biogenesis, motility, and plant immune elicitation. The transcriptional expression of *fliC-2* was significantly lower than that of *fliC-1* in medium and *in planta*, most likely due to variations in promoter activity. *In silico* prediction revealed that all *fliC-2* genes in the 12 Pseudomonas strains have a poorly conserved promoter motif. Compared to the Flg22-2 epitope (relative to FliC-2), Flg22-1 (relative to FliC-1) induced stronger FLAGELLIN SENSING 2 (FLS2)-mediated microbe-associated molecular pattern-triggered immunity and significantly inhibited plant root growth. A change in the 19th amino acid in Flg22-2 reduced its binding affinity to the FLS2/brassinosteroid insensitive 1-associated kinase 1 complex. Also, Flg22-2 epitopes in the other 11 Pseudomonas strains were presumed to have low binding affinity due to the same change in the 19th amino acid. These findings suggest that Pseudomonas has evolved duplicate flagellins, with only FliC-1 contributing to motility and plant immune elicitation.

**IMPORTANCE** Flagellins have emerged as important microbial patterns. This work focuses on flagellin duplication in some plant-associated Pseudomonas. Our findings on the divergence of duplicated flagellins provide a conceptual framework for better understanding the functional determinant flagellin and its peptide in multiple-flagellin plant-growth-promoting rhizobacteria.

## INTRODUCTION

Pseudomonas, a diverse and ecologically significant genus, is widespread in the natural environment. In addition to the opportunistic Pseudomonas pathogens, some rhizospheric or soil-dwelling Pseudomonas species act as plant-growth-promoting rhizobacteria (PGPR) that play substantial roles in plant growth promotion and disease suppression (1). Flagellum-mediated movement allows PGPR to move to nutrient-rich habitats and avoid conditions adverse to their survival. The bacterial flagellum is a macromolecular machinery with a rotary basal body embedded in the cell membrane, a curved hook, and one or more extracellular helical filaments that are composed of up to 30,000 flagellin monomers (2). As such, flagellin is required for bacterial motility, a fundamental function required for host colonization by pathogens, commensals, and symbionts (3). It is generally believed that the flagellar filament is encoded by a single flagellin gene per genome. However, as more and more bacterial genomes are sequenced, supernumerary flagellar loci have been discovered to be relatively common features in a broad taxonomic spectrum of bacteria, such as the order Enterobacterales, in which five (flag-1 to flag-5) flagellar loci occur on the genomes of enterobacterial taxa (4). It was reported that multiple flagellin genes had been found in more than 45% of the annotated bacterial genomes encoding flagella (5, 6), for reasons that are mostly unknown. The number of flagellin genes in such species usually ranges from two to seven, with a minority possessing more copies (5, 6).

Flagellar assembly and motility have been well studied in single-flagellin bacteria, such as Escherichia coli, which utilizes a single flagellin at any phase to assemble the filaments and control movement. However, the role of different flagellins in multiflagellin bacteria remains largely unclear. A few studies have demonstrated that a high degree of functional redundancy occurs in some bacteria, including Salmonella enterica serovar Typhimurium (7), Sinorhizobium meliloti (8 to 10), Bdellovibrio bacteriovorus (11), Helicobacter pylori (12), and *Vibrio* spp. (13 to 15), in which the flagellar filaments are assembled from all or part of the flagellins encoded in their genome. Loss of certain flagellins in these bacteria may result in changes in filament assembly and motility, depending on the species.

Regardless of whether the flagellar filament is simple or complex, the structures of most bacterial flagellin subunits are highly conserved in terms of amino-acid composition and subunit organization of the flagellin monomers. In a wide range of bacteria, flagellins comprise a ubiquitous microbe-associated molecular pattern (MAMP) that triggers both innate and adaptive immune responses in eukaryotic hosts and manipulates host–bacterial interactions (16). Flagellin 22 (Flg22), a 22-amino-acid peptide located in the highly conserved *N*-terminal region of flagellin, is perceived by the pattern recognition receptor FLAGELLIN-SENSING2 (FLS2) to induce immune reactions in various plants, such as tomato (Solanum lycopersicum), potato (Solanum tuberosum), tobacco (Nicotiana tabacum and N. benthamiana), and the model plant Arabidopsis thaliana (17). A recent high-throughput analysis showed that the Flg22 peptides of γ- and β-proteobacteria trigger strong oxidative bursts, whereas peptides from other (ε-, δ-, and α-) proteobacteria trigger a weak response, depending on the sequence divergence of the Flg22 epitopes in each taxonomic class (18). A massive screening of 412 Flg22 variants in the flagellin gene *fliC* of P. aeruginosa indicated that up to 80% of the variants could not restore motility of the immotile P. aeruginosa *fliC* mutant (19). Further analysis of FLS2-Flg22 variant interactions showed that more than 70% of variants retained interactions with FLS2 (19). Flg22 sequences from β- and γ-proteobacteria, *Bacillus*, and *Actinobacteria* resemble the immunogenic P. aeruginosa Flg22 sequence, whereas Flg22 sequences from *Rhizobiales* and *Caulobacterales* are substantially more divergent (20). These findings suggest that single amino-acid changes in the Flg22 epitope could affect bacterial motility and host–bacterial interaction. However, little is known about the biological roles and immunogenic and motility functions of multiple flagellins and natural Flg22 variants in a single bacterium.

Pseudomonas exhibits parasitic, commensal, and mutual interactions with host cells. Most Pseudomonas species, including the model pathogenic species P. aeruginosa and P. syringae, have only one *fliC* gene. In contrast, some commensal PGPR Pseudomonas strains, such as *P. kilonensis* F113 (previously P. fluorescens) and 1855-344, P. brassicacearum LBUM300, P. fluorescens et76, and Pseudomonas sp. CBZ-4, harbor an extra copy of the *fliC* gene (21). In *P. kilonensis* strain F113, a model fluorescent pseudomonad used to study secondary metabolite production and plant–bacterial interactions, a *fliC-1* mutant produced aflagellate bacterial cells, whereas a *fliC-2* mutant had a similar motility phenotype as the wild type (21). Reverse transcription (RT)-PCR analysis revealed that the *fliC-2* gene is not expressed in the wild-type strain F113, but is highly expressed in *kinB* and *algU* mutants, implying that the expression of different *fliC* copies may be influenced by environmental conditions (21). However, it is currently unknown whether the two *fliC* genes have redundancy in flagellar biogenesis and bacterial fitness or whether the two Flg22 epitopes differ in plant immune elicitation ability.

Here, phylogenetic analysis revealed that all FliC-1 and FliC-2 sequences from 12 Pseudomonas strains were clustered into two clades, and the evolutionary distances of the FliC-2 sequences were significantly shorter than those of the FliC-1. Knockout of *fliC-1*, but not *fliC-2*, impaired motility, flagellar biogenesis, plant immune elicitation, and root growth inhibition. We also demonstrated that when *fliC-1* is expressed in medium and *in planta*, *fliC-2* is sitting idle. Flg22-1 was more effective at eliciting plant immunity and inhibiting root growth than Flg22-2, mainly due to variation in the 19th amino-acid residue.

## RESULTS

### Characterization of the two flagellin genes in *P. kilonensis* F113.

**FIG 1:**
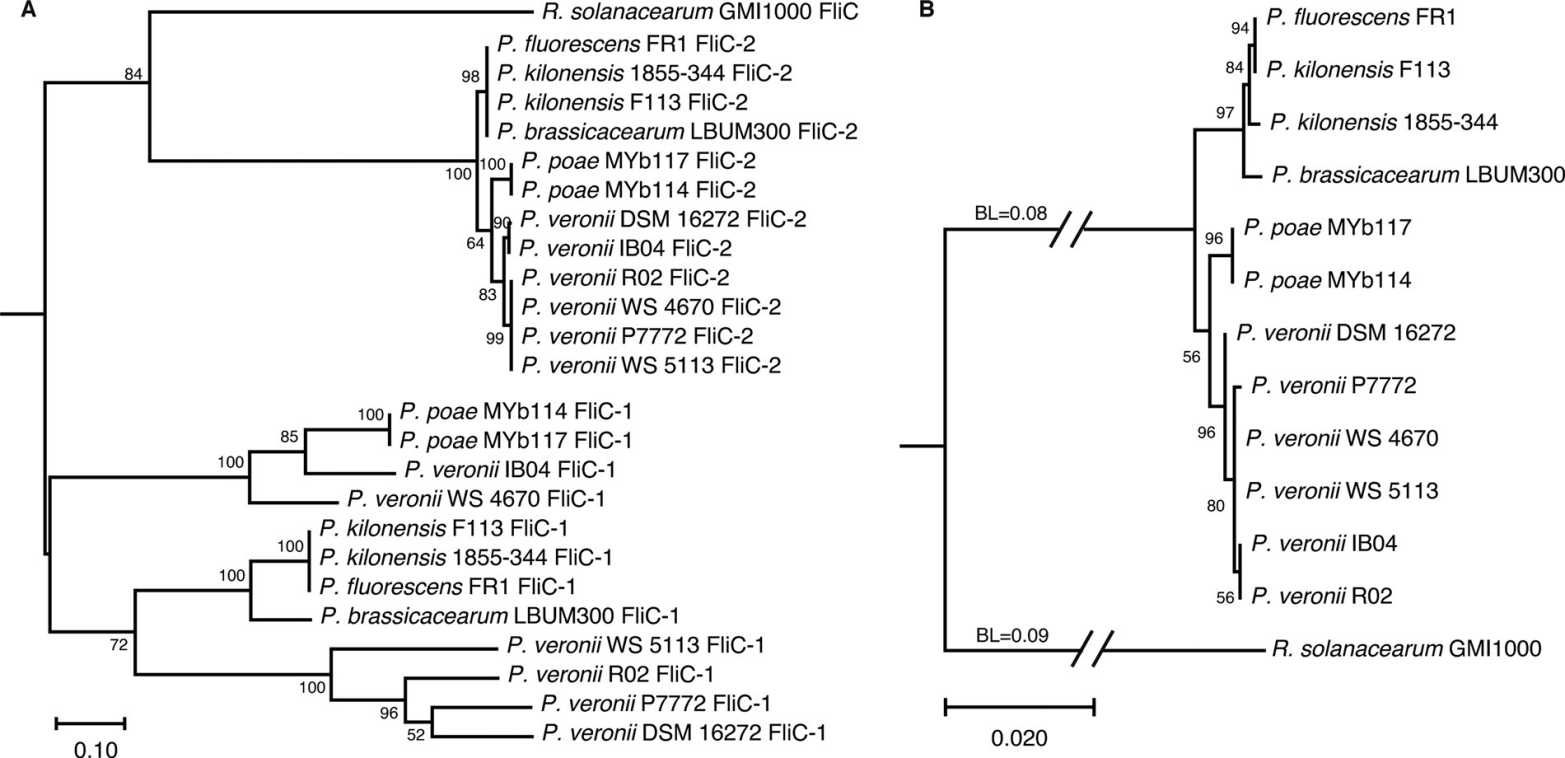
Phylogenetic analysis of flagellins of Pseudomonas strains. (A) Maximum-likelihood phylogeny based on protein sequence alignments of flagellins from 12 Pseudomonas strains and Ralstonia solanacearum GMI1000. The JTT matrix-based model with 500 bootstrap replicates was used. (B) Maximum-likelihood phylogeny based on 16S rRNA gene sequences from 12 Pseudomonas strains and Ralstonia solanacearum GMI1000. The Tamura–Nei model with 500 bootstrap replicates was used. 16S rRNA gene sequences and flagellin sequences from *R. solanacearum* GMI1000 were regarded as an outgroup. The numbers at the branches represent the confidence levels of the taxa clustered in the tree. The scale bar reflects evolutionary distance. BL, branch lengths.

*P. kilonensis* strain F113 possesses two flagellin-encoding genes, *fliC-1* (PSF113_1554) and *fliC-2* (PSF113_0740), which are located in two separate flagellar clusters (21). The FliC-1 and FliC-2 proteins comprise 283 amino acids and 350 amino acids, respectively, which show 45.04% identity (Fig. S1). We performed a comprehensive BLAST analysis (Expect threshold was 0.05) to search for FliC-2 homologs in 967 genomes of the P. fluorescens group (taxid:136843) in NCBI, and 11 sequences were collected from P. veronii R02, *P. veronii* WS 4670, *P. veronii* WS 5113, *P. veronii* P7772, *P. veronii* IB04, *P. veronii* DSM 16272, P. fluorescens FR1, *P. kilonensis* 1855-344, *P. brassicacearum* LBUM300, *P. poae* MYb114, and P. poae MYb117. Phylogenetic analysis using MEGA X revealed that all FliC-1 and FliC-2 sequences clustered divergently into two clades (Fig. 1A), suggesting that these two kinds of flagellins (*fliC-1* and *fliC-2* in 12 Pseudomonas strains, respectively) might evolve independently. However, the evolutionary distances of the FliC-2 sequences were significantly shorter than those of the FliC-1 sequences. In addition, the six *P. veronii* strains in the FliC-1 clade were separated by a subgroup of *P. kilonensis* F113, FR1, 1855-344, and P. brassicacearum LBUM300. Conversely, *P. veronii* strains in the FliC-2 clade were clustered together. Although the taxonomic status of some collected strains remains to be determined, the FilC-2 phylogenetic tree seems to be more identical to the tree of the 16S rRNA genes (Fig. 1B), suggesting that FliC-2 is more appropriate than FliC-1 to illustrate the phylogenetic relationships among the collected 12 strains from the P. fluorescens complex (22).

### A *fliC-1* mutant is deficient in motility and flagellar biogenesis.

**FIG 2:**
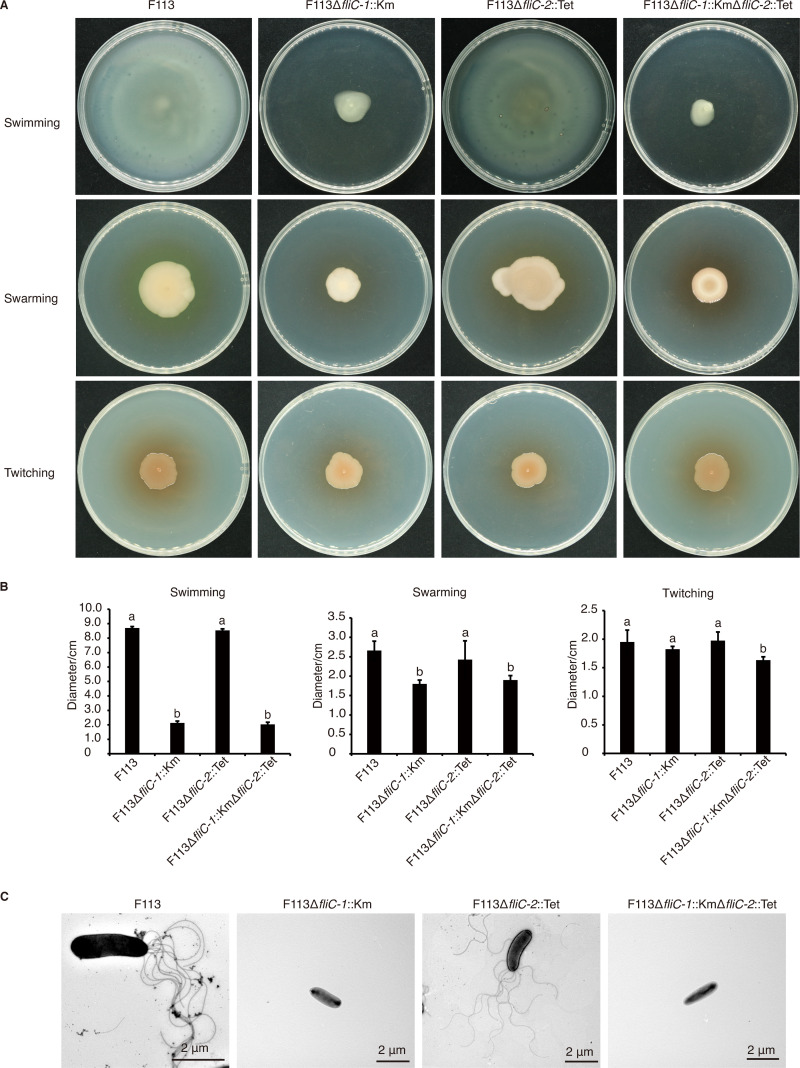
Motility and flagellar examination of *P. kilonensis* F113 and its *fliC* mutants. (A) Swimming, swarming, and twitching motilities on KB swim plates (0.3%, 0.5%, and 1.0% agar, respectively). Photographs were taken 24 h (swimming), 48 h (swarming), or 96 h (twitching) after incubation. (B) The quantitative analysis of swimming, swarming, and twitching abilities of *P. kilonensis* F113 and the *fliC* mutants. Different letters indicate statistically significant differences between different treatments (one-way ANOVA, Tukey’s test; *P < *0.05). (C) Transmission electron microscopy images of the flagella of *P. kilonensis* F113 and its derivatives. All of the experiments were repeated three times with similar results.

Bacteria deploy the flagellar apparatus as a power motor to change their motility patterns in order to occupy a more suitable habitat. To investigate which flagellin is required for motility and flagellar biogenesis, we generated mutants of the two flagellin genes. Strain F113 showed strong swimming and swarming activities on agar plates and formed expanding circular colonies after 24 h of incubation. The F113Δ*fliC-1*::Km mutant and F113Δ*fliC-1*::KmΔ*fliC-2*::Tet double mutant were severely compromised in motility and formed significantly smaller colonies than the wild-type strain, whereas the *fliC-2* mutation had no effect on swimming or swarming (Fig. 2A and B). Also, complemented with *fliC-1* under the native promoter to F113Δ*fliC-1*::Km resulted in the recovery of swimming ability, whereas complemented with *fliC-2* under the native promoter to F113Δ*fliC-1*::KmΔ*fliC-2*::Tet did not (Fig. S2A and B). None of the single *fliC* mutants were affected in twitching motility (Fig. 2A and B). Morphological observation under a transmission electron microscope indicated that the wild-type strain F113 possessed one or more (up to 9) polar flagella (Fig. 2C and Fig. S2C). However, rather than the *fliC-2* single mutant (F113Δ*fliC-2*::Tet), the *fliC-1* single mutant (F113Δ*fliC-1*::Km) and the *fliC-1* and *fliC-2* double mutant (F113Δ*fliC-1*::KmΔ*fliC-2*::Tet) were deficient in flagella production (Fig. 2C and Fig. S2C). These findings imply that *fliC-1*, rather than *fliC-2*, is essential for flagellar biogenesis, bacterial swimming, and swarming motility in strain F113 under laboratory conditions.

### The *fliC-1* mutant fails to stimulate plant immunity and affect root growth.

**FIG 3:**
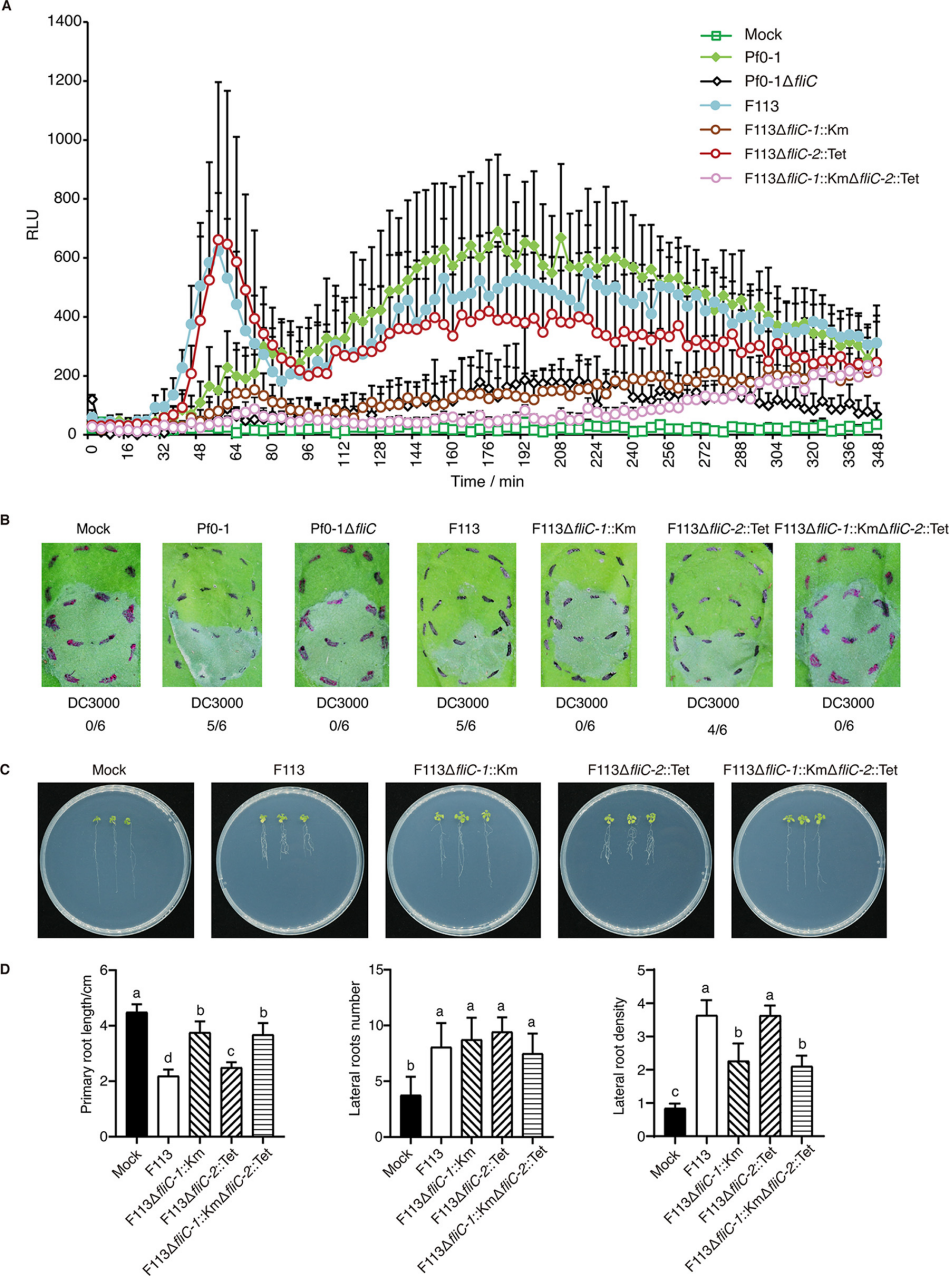
Contribution of the duplicated flagellins to plant immunity and root growth. (A) ROS induced by P. fluorescens Pf0-1, *P. kilonensis* F113, and the *fliC* mutants in N. benthamiana (RLU, relative light unit). (B) Challenge-inoculation HR assays for functional PTI were conducted by first infiltrating N. benthamiana leaves with 1 × 10^8^ CFU/mL of the test Pseudomonas strains (upper circles). After 6 h, an overlapping inoculation of 5 × 10^6^ CFU/mL of the HR-inducing strain *Pst* DC3000 (lower circles) was made. The fraction under each image indicates the number of times that the HR was inhibited compared to the number of test inoculations. (C) Effects of *P. kilonensis* F113 and its *fliC* mutants on shoot and root growth in *A. thaliana* accession Col-0. (D) Quantitative analysis of primary root length, number of lateral roots, and lateral root density. Different letters indicate statistically significant differences between different treatments (one-way ANOVA, Tukey’s test; *P < *0.05). All experiments were repeated three times with similar results.

Flagellin derived from bacterial flagella is recognized as a typical MAMP perceived by the plant receptor FLS2 and stimulates plant innate immunity (17, 23). To assess whether the two *fliC* genes are involved in eliciting pattern-triggered immunity (PTI) responses, we first examined the ROS production induced in N. benthamiana by strain F113 and its *fliC* mutants. P. fluorescens Pf0-1 and its flagellin mutant Pf0-1Δ*fliC* were employed as positive and negative control, respectively (24). Strains F113 and F113Δ*fliC-2*::Tet stimulated similarly strong ROS bursts in tobacco leaves, as did the positive control P. fluorescens Pf0-1; however, no ROS bursts were observed after treatments with F113Δ*fliC-1*::Km, F113Δ*fliC-1*::KmΔ*fliC-2*::Tet, and the negative control Pf0-1Δ*fliC* (Fig. 3A). Furthermore, challenge inoculation of strains F113 and F113Δ*fliC-*2::Tet compromised *Pst* DC3000-induced hypersensitive response (HR) in N. benthamiana, whereas the F113Δ*fliC-1*::Km and F113Δ*fliC-1*::KmΔ*fliC-2*::Tet mutants failed to inhibit *Pst* DC3000-induced HR (Fig. 3B). These results were further confirmed by electrolyte leakage data (Fig. S3A), indicating that FliC-1, but not FliC-2, contributes to PTI activation.

We next explored the effects of the mutants on *Arabidopsis* root growth. Compared to mock inoculation with ddH_2_O, strain F113 and all mutants significantly inhibited primary root growth. However, F113 and F113Δ*fliC-2*::Tet showed significantly stronger inhibitory abilities than F113Δ*fliC-1*::Km and F113Δ*fliC-1*::KmΔ*fliC-2*::Tet (Fig. 3C and D). Further, strain F113 and all mutants significantly increased the number of lateral roots compared with the mock treatment, but there was no significant difference among the strains (Fig. 3C and D). It is noteworthy that lateral root density was significantly increased upon treatment with F113, whereas F113Δ*fliC-1*::Km, but not F113Δ*fliC-2*::Tet, strongly reduced lateral root density (Fig. 3C and D). Taken together, these data suggested that FliC-1 plays a major role in stimulating plant immunity and inhibiting root growth.

### Transcriptional expression of *fliC-1* is significantly higher than that of *fliC-2* in medium and *in planta*.

**FIG 4:**
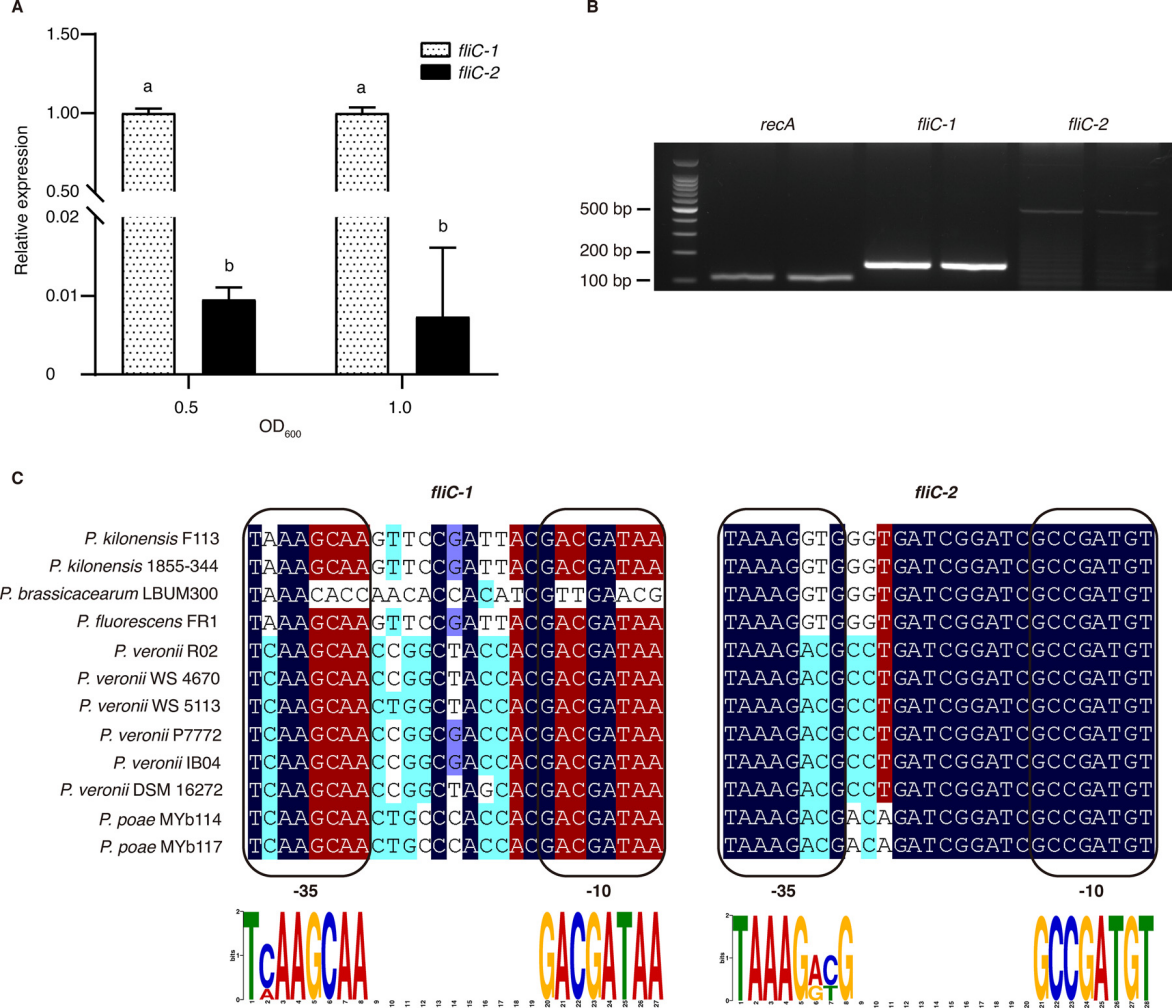
*fliC* gene expression and promoter prediction. (A) RT-qPCR analysis of *fliC-1* and *fliC-2* expression in *P. kilonensis* F113 cultured in KB medium. (B) RT-PCR of *fliC-1* and *fliC-2* expression in *P. kilonensis* F113 infiltrated into N. benthamiana. (C) Alignment of the *fliC-1* and *fliC-2* promoter regions. The -10 and -35 regions for each promoter are indicated in boxes. Dark blue indicates 100% identity, red indicates ≥75% identity, cyan indicates ≥50% identity, and light blue indicates ≥33% identity. The height of each letter in the MEME LOGO represents the relative frequency of each base at different positions in the consensus sequence. Different letters indicate statistically significant differences between different treatments (one-way ANOVA, Tukey’s test; *P < *0.05). All experiments were repeated three times with similar results.

To investigate whether gene expression underlies the divergent functions of the two flagellin genes, we further examined the transcriptional expression of the two genes in medium and *in planta*. RT-qPCR results showed that *fliC-1* was significantly more strongly expressed than *fliC-2* in King’s medium B (KB) in both the logarithmic and stationary phases (Fig. 4A). Furthermore, RT-PCR assays demonstrated that *fliC-2* expression was lower than that of the control *recA*, whereas *fliC-1* expression was significantly higher than that of *fliC-2* and *recA* (Fig. 4B; Fig. S3C). These results suggested that the two *fliC* genes show a significantly different transcriptional expression and may lead to divergent biological functions.

The expression of the flagellar system is regulated by a complex hierarchy (25, 26). σ^28^ is a key master regulator that binds to a specific promoter region of the *fliC* genes (27, 28). To determine the promoters of the two *fliC* genes, we collected 13 well-studied *fliC* promoters from various bacteria (Table S5) and generated a *fliC* promoter diagram (Fig. S4A). Using this motif, we scanned the promoter regions of the two *fliC* genes of strain F113. The results demonstrated that *fliC-1* has a conserved promoter (*P = *1.88E-06), whereas the promoter of *fliC-2* is more variable (*P = *6.45E-06) (Fig. 4C; Table S6). We speculated that sequence polymorphism in the promoters may affect the expression levels of *fliC-1* and *fliC-2*. Given the differential conservation of the two *fliC* promoters in strain F113, we asked whether the other 11 strains with two *fliC* genes would have the same characteristics. We performed promoter scanning and sequence alignments of all *fliC-1* and *fliC-2* genes (Fig. 4C). Notably, we observed that all *fliC-1* promoters were more conserved than *fliC-2* promoters, except in P. brassicacearum LBUM300, which has an untypical *fliC-1* promoter (Fig. 4C). To test whether the promoter of *fliC-1* could drive the expression of *fliC-2*, we developed a hybrid construct to quantify the transcription of *fliC-2*. Notably, the expression level of *fliC-2* driven by *the fliC-1* promoter in the double mutant F113Δ*fliC-1*::KmΔ*fliC-2*::Tet was significantly increased (Fig. S4B). We then asked whether highly expressed *fliC-2* could recover swimming motility or PTI elicitation in F113Δ*fliC-1*::KmΔ*fliC-2*::Tet. However, swimming activity and ETI-cell death suppression were not observed after complementation with the *fliC-2* gene driven by the *fliC-1* promoter (Fig. S2A and D). Taken together, these findings imply that differential promoter activity results in divergent transcriptional expression of duplicated *fliC* genes in *P. kilonensis* F113. Although *fliC-2* has low biological expression, artificially high expression of it cannot perform biological functions such as swimming motility and PTI elicitation.

### Flg22-1 induces stronger plant immune responses than Flg22-2.

**FIG 5:**
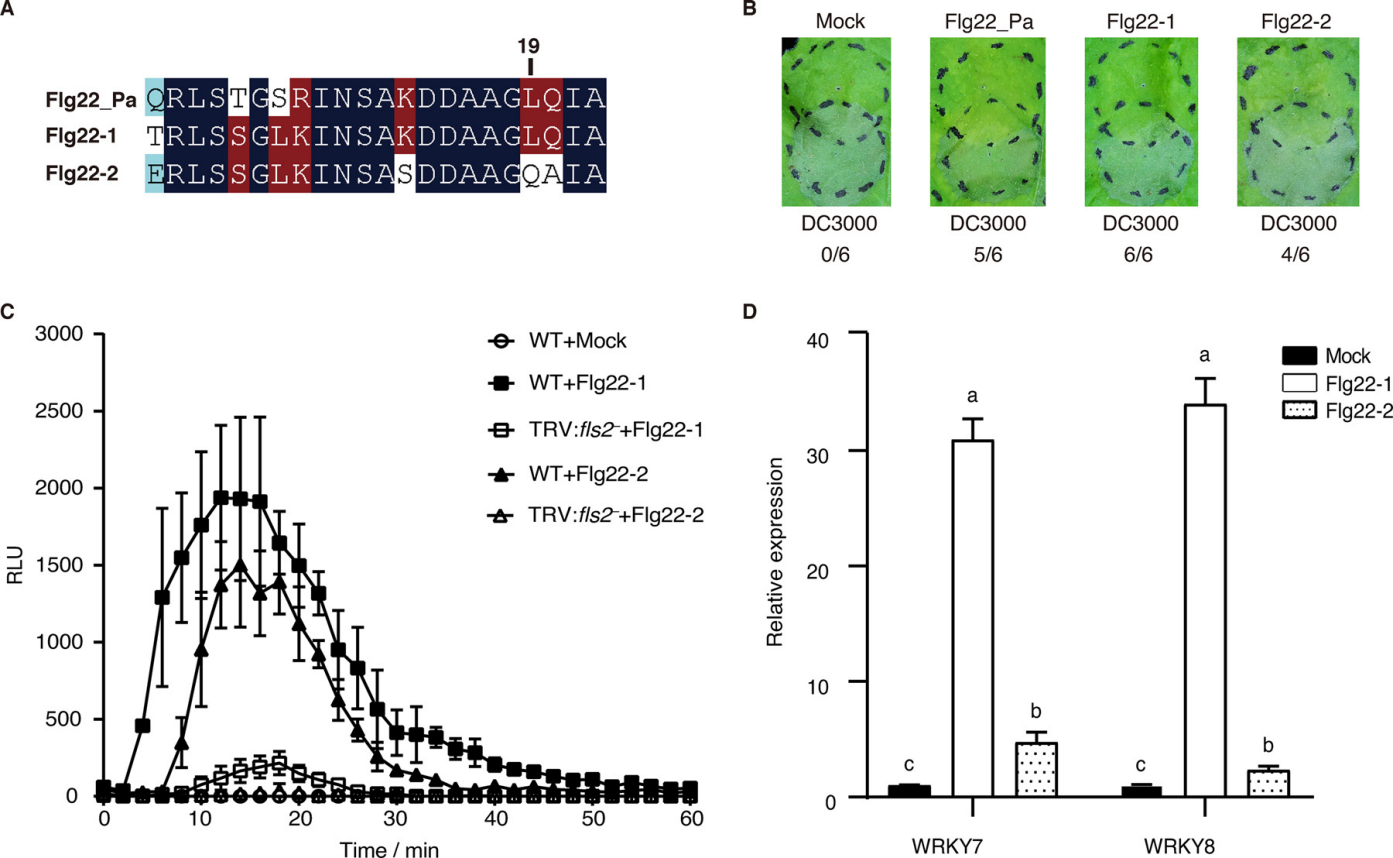
Plant immunity induced by Flg22-1 and Flg22-2. (A) Sequence alignment of Flg22_Pa from P. aeruginosa PAO1 and Flg22-1, and Flg22-2 from *P. kilonensis* F113. Dark blue indicates 100% identity, red indicates ≥50% identity, and light blue indicates ≥33% identity. (B) Challenge-inoculation HR assays for functional PTI were conducted by first infiltrating N. benthamiana leaves with 10 μM Flg22 peptides (upper circles). After 6 h, an overlapping inoculation of 5 × 10^6^ CFU/mL of the HR-inducing strain *Pst* DC3000 (lower circles) was made. The fraction under each image indicates the number of times that the HR was inhibited to the number of test inoculations. (C) ROS induced by 0.1 μM Flg22 peptides in wild-type and *fls2*-silenced N. benthamiana plants. (D) PTI marker gene expression induced by 1 μM Flg22 peptides in N. benthamiana. Different letters indicate statistically significant differences between different treatments (one-way ANOVA, Tukey’s test; *P < *0.05). All of the experiments were repeated three times with similar results.

The Flg22 region in the bacterial flagellin N terminus carries the innate immune elicitation determinant that is recognized by FLS2 in many plant species (17). To examine the Flg22 peptides of the two flagellins in strain F113, we extracted the sequences of Flg22-1 and Flg22-2 and aligned them with the commercial Flg22_Pa (Fig. 5A). The results showed that Flg22-1 and Flg22-2 have 18 and 15 amino-acid residues identical to Flg22_Pa, respectively. Asp^14^, Asp^15^, Leu^19^, and Ile^21^ are key residues of Flg22_Pa for plant immunity elicitation (29). In strain F113, Flg22-1 has all four conserved amino acids, whereas Flg22-2 only has three; Leu^19^ is replaced by Gln. We synthesized Flg22-1 and Flg22-2 and examined their potential in stimulating plant immunity. Inoculation of Flg22-1 resulted in full inhibition of the HR triggered by *Pst* DC3000, whereas Flg22-2 exhibited moderate HR inhibition ability (Fig. 5B). ROS assays indicated that Flg22-1 induced stronger ROS production than Flg22-2 (Fig. 5C and Fig. S5). Notably, neither of the two peptides induced ROS production in *fls2*-silenced N. benthamiana (Fig. 5C and Fig. S5), suggesting that Flg22-1 and Flg22-2 are both sensed by FLS2. Further, both Flg22-1 and Flg22-2 induced callose deposition, but Flg22-1 induced more deposits than Flg22-2 (Fig. S6). Both Flg22-1 and Flg22-2 induced the expression of two PTI marker genes, *WRKY7* and *WRKY8*, whereas Flg22-1 induced significantly higher levels of defense gene expression than Flg22-2 (Fig. 5D). Taken together, these results indicated that both Flg22-1 and Flg22-2 can induce plant immune responses, but Flg22-1 is more potent than Flg22-2.

### Flg22-1 inhibits plant growth more robustly than Flg22-2.

**FIG 6:**
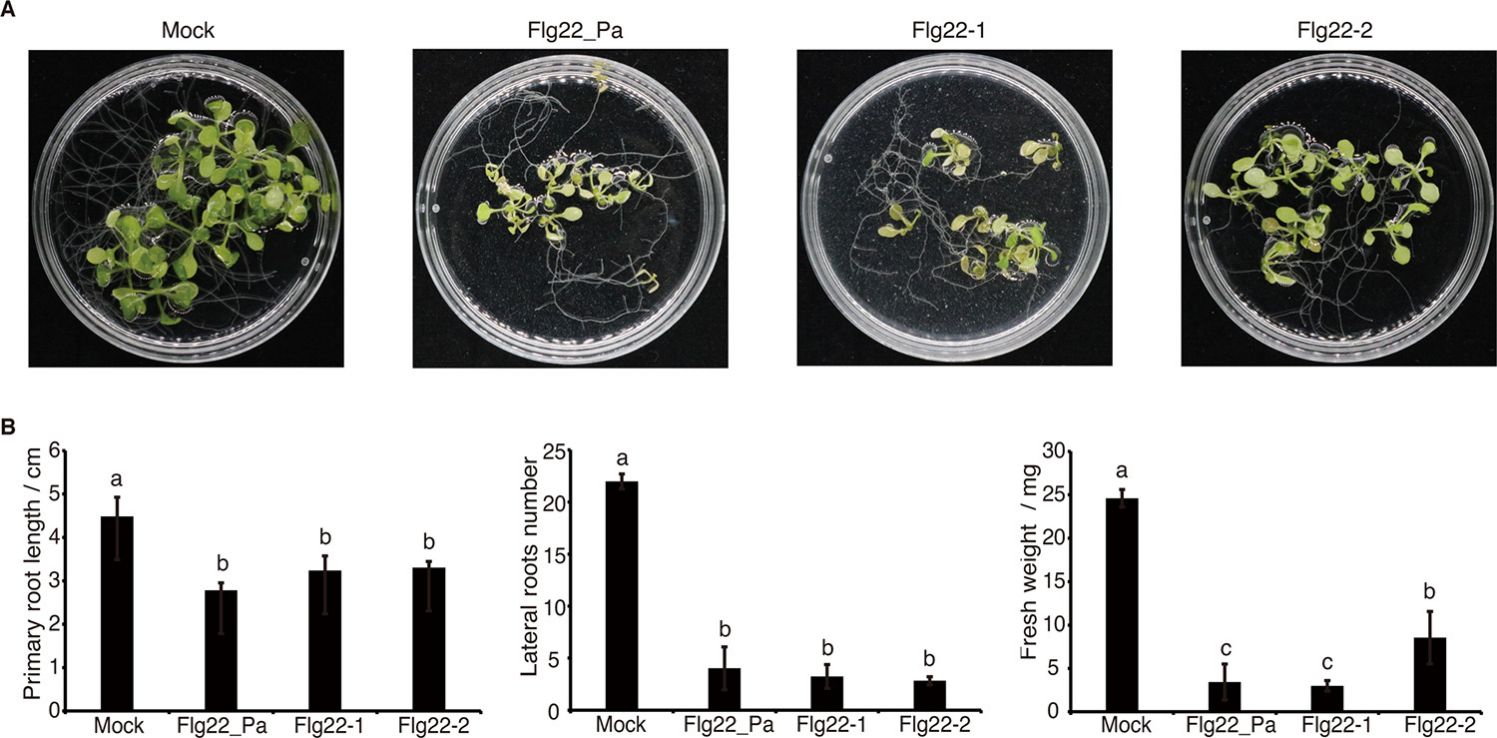
Effects of Flg22 peptides on plant growth. (A) Phenotypes of *A. thaliana* seedlings treated with Flg22_Pa, Flg22-1, and Flg22-2. (B) Quantitative analysis of primary root length, number of lateral roots formed, and fresh weight. Different letters indicate statistically significant differences between different treatments (one-way ANOVA, Tukey’s test; *P < *0.05). All experiments were repeated three times with similar results.

We next investigated whether the peptides Flg22-1 and Flg22-2 could inhibit plant growth. After 12 days, leaves from seedlings treated with Flg22_Pa and Flg22-1 developed significantly more chlorosis than those from plants treated with Flg22-2 (Fig. 6A and Fig. S7). Primary root lengths in the Flg22_Pa, Flg22-1, and Flg22-2 treatments were significantly shorter than those in the MS control, and lateral root numbers were reduced by approximately four times. Moreover, the fresh weights of whole plants in the Flg22-Pa, Flg22-1, and Flg22-2 treatments were reduced by approximately three to five times compared to those in the MS control, of which Flg22-2 showed weaker inhibition than Flg22_Pa and Flg22-1 (Fig. 6B).

### The 19th residue of Flg22 is predicted to be critical for receptor interaction.

Given the differential ability of Flg22-1 and Flg22-2 to stimulate plant immunity and inhibit plant growth, we examined the key amino acids involved in receptor recognition. The binding affinity of Flg22 with FLS2 and BAK1 was predicted by *in silico* modeling using the MutaBind2 pipeline. Flg22-1 was predicted to bind the FLS2/BAK1 receptor complex with high affinity (ΔΔG_bind_ of Flg22-1-FLS2 = −0.59 kcal/mol; Flg22-1-FLS2-BAK1 = −1.12 kcal/mol) (Table 1). In contrast, seven of the Flg22-2 residues differed from those of Flg22_Pa, and a significant reduction in binding was predicted for FLS2 (ΔΔG_bind_ = 2.2 kcal/mol), whereas a moderate reduction was predicted for BAK1 (ΔΔG_bind_ = 0.16 kcal/mol) (Table 1). To determine the critical residues, we performed single amino-acid replacements on Flg22_Pa based on the Flg22-2 sequence (Table 2). The predictions showed that K13S and Q20A in Flg22_Pa might have reduced the binding stability with FLS2 significantly. In contrast, L19Q in Flg22_Pa might have affected FLS2-Flg22-BAK1 complex formation significantly. It has been reported that Leu^19^ in Flg22 is the only contact site for binding to FLS2-BAK1 (20). Thus, mutation of the 19th amino acid in Flg22-2 might be the primary cause of reduced plant immunity and growth inhibition. Finally, we examined the identities of other Flg22-1 and Flg22-2 epitopes in the other 11 strains. All Flg22-2 sequences showed 100% identity to that of strain F113, whereas the Flg22-1 sequences were variable (Fig. S8). Remarkably, *P. veronii* strains showed significant variation; five contained Met rather than Leu at the 19th position. We assessed the binding affinities of the other Flg22-1 peptides with FLS2 and BAK1 using the MutaBind2 pipeline. Although some strains had a variable Flg22-1, none of them showed a significant change in binding affinity (Table S3, S4). These results suggested that Flg22-1 and Flg22-2 in all of the tested strains might have divergent effects on PTI stimulation.

**TABLE 1:** Binding affinity between Flg22 and FLS2 or BAK1

Peptide	Sequence^*a*^	Phase 1 (FLS2-Flg22) ΔΔG_bind_ (kcal mol^−1^)^*b*^	Phase 2 (FLS2/Flg22-BAK1) ΔΔG_bind_ (kcal mol^−1^)
Flg22-1	**T**RLS**S**G**LK**IN SAKDDAAGLQIA	−0.59	−1.12
Flg22-2	**E**RLS**S**G**LK**IN SA**S**DDAAG**QA**IA	2.2	0.16
Flg22_Pa	QRLSTGSRIN SAKDDAAGLQIA		

a Letters in bold indicate the mutated residues compared to Flg22_Pa.

b ΔΔG_bind_ (kcal mol^−1^): a positive value indicates a destabilizing mutation and a negative value a stabilizing mutation.

**TABLE 2:** Binding affinity between Flg22 with a single mutation and FLS2 or BAK1

Peptide	Mutated residue	Phase 1 (FLS2-Flg22)		Phase 2 (FLS2/Flg22-BAK1)	
		ΔΔG_bind_ (kcal mol^−1^)	Deleterious^*a*^	ΔΔG_bind_ (kcal mol^−1^)	Deleterious^*a*^
Flg22-2	Q1E	1.38	No	−0.44	No
	T5S	0.72	No	−0.65	No
	S7L	−0.13	No	−1.17	No
	R8K	1.37	No	−0.36	No
	K13S	2.22	Yes	–0.3	No
	L19Q	1.37	No	1.52	Yes
	Q20A	2.09	Yes	−0.06	No

a Deleterious (yes/no): the MutaBind2 server uses ΔΔG ≥ 1.5 or ≤−1.5 kcal mol^−1^ to define whether or not a mutation is deleterious. ΔΔG ≥ 1.5 kcal mol^−1^ is deleterious.

## DISCUSSION

Many bacterial species employ flagella to move to and colonize their preferred environmental niche. With the increase in genome sequences, multiple flagellin genes are increasingly being found in bacteria. However, the functions of multiple flagellins in bacteria remain largely elusive. Here, we demonstrated that the two flagellin proteins of *P. kilonensis* F113 are divergent evolutionarily, with FliC-1 functioning as the major determinant of motility and plant immunity stimulation. Notably, mutation of the 19th amino acid may have been a significant cause of reduced binding affinity with FLS2/BAK1 and plant immunity, as predicted by affinity prediction.

We found that FliC-1 and FliC-2 of *P. kilonensis* F113 show 45% identity, mostly in the two termini (Fig. S1). The middle region of FliC-1 of strain F113 is variable and 67 amino acids shorter than that of FliC-2 (Fig. S1). This was also observed for the 11 FliC-1 homologs from various Pseudomonas strains (data not shown). It has been reported that the conserved N- and C-terminal regions of flagellin proteins mediate filament assembly and harbor the hot spot recognized by the host immunity receptor, whereas the variable central region contains the surface-exposed D2-D3 domains (30 to 32). Although a previous study predicted that the second flagellar system of *P. kilonensis* F113 containing FliC-2 resulted from an insertion event based on a syntenic comparison of the genomes of strain F113 and P. brassicacearum NFM421 (21), the origin of FliC-2 is still unknown. Genome analysis of a broader range of related strains and a detailed phylogenetic description of relationships among Pseudomonas species are required to investigate the origin and selection rates of FliC-2 and to assess the extent of lateral gene transfer and recombination within these loci.

*P. kilonensis* F113 produces polar flagella, but only FliC-1 is required for flagellar assembly and mobility (Fig. 2) under laboratory conditions. These findings are consistent with those in Proteus mirabilis, in which *flaA* and *flaB* encode two flagellin proteins, but only FlaA is involved in filament assembly (33). However, a few studies showed that some bacteria deploy multiple flagellin proteins to build the flagellar complex (11, 33 to 35). B. bacteriovorus has six flagellin genes, *fliC-1*–*6*, all of which except *fliC4*, which expressed at a low level, synergistically encode the single polar flagellum (11). Notably, we discovered that *P. kilonensis* F113 produces 1 to 9 polar flagella, which differs from the single polar flagellum reported in a previous study (21). It has been suggested that polar flagella, as well as peritrichous flagella, are induced in solid medium or medium with a high viscosity (36, 37). Although we did not assess what determines the polar flagella number in *P. kilonensis* F113, further research into medium composition, culture conditions, and gene regulation would shed light on the mechanisms of flagella production.

We found that in *P. kilonensis* F113, *fliC-1* is significantly more strongly expressed than *fliC-2*, which is largely dependent on the promoter activity. Notably, the *fliC-1* promoter is more conserved than that of *fliC-2* compared with the typical promoter regulated by the transcriptional regulator σ^28^. The same characteristics were found in the other Pseudomonas strains containing duplicated *fliC* genes. Interestingly, it has been reported that two flagellin genes in many Salmonella strains, *fliC* and *fljB*, encode phase 1 and phase 2 flagellins, respectively, which are not expressed simultaneously. The expression of the two loci is governed by a switch mechanism that is regulated by the invertible element *hin*, which appears to be unique to Salmonella (37). Given this, the second flagellin FljB is considered a genetic “spare tire” used in particular environmental circumstances that is used less often than FliC and is less critical to survival (38). Although it is unknown whether *P. kilonensis* F113 and its relatives are diphasic and capable of phase variation, the soil-dwelling and plant-colonizing properties of Pseudomonas imply that the second FliC could be used in unpredictable short-term emergencies, such as adapting to new niches, avoiding protozoan predation, and immunologic escape.

The FLS2/BAK1 complex is the direct receptor of Flg22 (19, 20, 29). The 19th amino acid, Leu, is the only residue that interacts with FLS2 and BAK1 and is located in the “message” area, which is involved in immune response activation (23, 29, 39). In all of the strains in this study, the 19th amino acid of Flg22-2 was Gln rather than Leu, resulting in reduced binding affinity with FLS2/BAK1. Some differences at the 19th amino acid of Flg22 have also been found in S. meliloti, A. tumefaciens, *Candidatus* Liberibacter solanacearum, and *Candidatus* Liberibacter asiaticus (40, 41), whose flagellins are nonimmunogenic, leading to immune evasion. However, a few studies have shown that replacements of Leu^19^ constrain motility and immunogenicity (19). For instance, lack of Leu^19^ in Flg22_Pa dramatically decreased plant immune activity (17, 23), and removal of Leu^19^ in Flg15-Δ3 even changed its biological activity into an antagonist (23). Interestingly, we found that some Pseudomonas strains had Met rather than Leu at the 19th residue of Flg22-1, which did not affect the binding affinity with FLS2/BAK1 (Table S3, S4). A recent study showed that Met at the 19th position of Flg22^5013^ had no adverse effect on motility or FLS2 interaction (20). Previous and our findings combined fully explain the importance of Leu^19^ in Flg22, and this residue is functionally equivalent to Met, but not Gln. In conclusion, we hypothesize that the divergence of the two flagellins in *P. kilonensis* F113 and other strains with duplicated flagellins was driven by selection to evade plant immune detection. Our findings on the divergence of duplicated flagellins provide a conceptual framework for better understanding the functional determinant flagellin and its peptide in multiple-flagellin PGPRs.

## MATERIALS AND METHODS

### Bacterial strains and plasmids.

The bacterial strains and plasmids used in this study are listed in Table S1. Pseudomonas fluorescens Pf0-1, Pf0-1Δ*fliC*, P. syringae pv. *tomato* (*Pst*) DC3000, *P. kilonensis* F113, and the *fliC* mutants were grown in King’s medium B (KB) at 28°C (24, 42, 43). Escherichia coli (DH5α) was grown in Luria-Bertani (LB) broth at 37°C. To propagate plasmids and select transformants, antibiotics were added at the following final concentrations: 100 μg/mL ampicillin, 50 μg/mL kanamycin, and 20 μg/mL tetracycline, as required (44). pT18*mob* is a derivative of pT18*mobsacB* (24), in which the *sacB* gene fragment was removed by digestion with *Aha* III in this study.

### Construction of *fliC* mutants and complemented strains.

To construct *P. kilonensis* F113Δ*fliC-1*::Km, a 509-bp fragment of the *fliC-1* gene was amplified from *P. kilonensis* F113 using primers WHL1048 and WHL1049 and cloned into p2P24 (45). The final construct was transformed into *P. kilonensis* F113 by triparental mating. To construct *P. kilonensis* F113Δ*fliC-2*::Tet and *P. kilonensis* F113Δ*fliC-1*::KmΔ*fliC-2*::Tet, a 634-bp fragment of the *fliC-2* gene was amplified from *P. kilonensis* F113 using primers WHL1104 and WHL1105 and cloned into pT18mob. Triparental mating was used to introduce the vector into *P. kilonensis* F113 and the *fliC-1* mutant. The primers used in this study are listed in Table S2. The complemented strains are created through triparental mating of pBBR1-MCS5-C1, pBBR1-MCS5-C2, and pBBR1-MCS5-C3.

### Transmission electron microscopy and motility assays.

*P. kilonensis* F113 and derivatives were grown in KB medium for 24 h. Grids were stained, and photographs were taken as described previously (46). Swimming, swarming, and twitching motility were tested on KB media with 0.3%, 0.5% (plus 5 g/L glucose), and 1% agar according to a previous report (46). The plates were photographed after 24 h, and colony diameters were measured for quantitative analysis. All assays were repeated at least three times, with multiple replicates.

### Reactive oxygen species (ROS) assay.

Bacterial suspensions at OD_600_ = 0.5 (5 × 10^8^ CFU/mL) were infiltrated into *N. benthamiana* leaves. P. fluorescens Pf0-1 and Pf0-1Δ*fliC* were used as positive and negative controls, respectively. MgCl_2_ (10 mM) was used as a mock control. Six hours after infiltration, leaf disks (0.5 cm diameter) were punched out and soaked in 100 μL of 0.5 mM L-012 (Wako, Japan). The intensity of ROS production was determined by monitoring the chemiluminescence using a Tecan microplate reader (Tecan, Switzerland) (24).

For peptide-triggered ROS assays, N. benthamiana leaf discs were soaked in 100 μL of distilled water for 12 hours. Water was replaced with 100 μL of a solution containing 34 mg/mL of luminol, 10 μg/mL of horseradish peroxidase, and 0.1 μM Flg22. Luminescence was measured as described above (47). All assays were repeated at least three times, with multiple replicates.

### Callose deposition assay.

Callose deposition in N. benthamiana was assayed as described previously (47), with slight adjustments. The leaves of N. benthamiana plants were infiltrated with 1 μM Flg22 peptide derived from P. aeruginosa (Flg22_Pa), Flg22-1, or Flg22-2. Sterile water was used as a mock control. Leaf disks (1 cm diameter) collected 6 h after infiltration were floated in 2 mL of 95% ethanol and incubated for decolorization at 37°C for 6 h until completely transparent (the decolorization time could be shortened to less than 4 h by refreshing the ethanol half-way the incubation). The decolorized leaves were washed twice with 70% ethanol and then thrice with sterile water. Aniline blue solution (1%, dissolved in 150 mM K_2_HPO_4_, adjusted to pH 9.5 with KOH) was added, and the leaf disks were placed in a dark environment for 1 h. Callose deposits were visualized using a confocal microscope (LSM 880, Zeiss). The assays were repeated at least three times, with multiple replicates.

### Challenge-inoculation hypersensitive response (HR) assay.

Bacterial suspensions at OD_600_ = 0.1 (1 × 10^8^ CFU/mL) or peptides at 10 μM were infiltrated into *N. benthamiana* leaves. After 6 h, the bacterial pathogen *Pst* DC3000 was inoculated into the prior infiltration area at OD_600_ = 0.005 (5 × 10^6^ CFU/mL). After 2 days postinoculation (dpi), cell death was measured and photographs acquired. An ion leakage assay was conducted as previously reported (48). The assay was repeated at least three times, with multiple replicates.

### Seedling growth inhibition assay.

Seeds of Arabidopsis thaliana Col-0 were surface-sterilized with 70% ethanol for 5 min and rinsed with sterile water thrice to remove the ethanol. The sterilized seeds were sown on 1× Murashige and Skoog (MS) plates supplemented with 0.5% sucrose and adjusted to pH 5.7 with KOH (49). Seedings were stratified at 4°C in the dark for 2 days and transferred to a growth chamber (21 to 23°C; 16 h light, 8 h dark; light intensity 100 μmol m^−2^ s^−1^) (50). *P. kilonensis* F113 and derivatives at 1 × 10^8^ CFU/mL or peptides at 10 μM were dipped onto the root tips. Primary root length, number of lateral roots, and shoot fresh weight were measured at 14 dpi (51). Lateral root density was calculated by dividing the number of lateral roots on the primary root by root length as reported previously (52). The assay was repeated at least three times with multiple replicates.

### Virus-induced gene silencing (VIGS) in N. benthamiana.

N. benthamiana plants were grown in a chamber with 16 h light/8 h dark, 60% humidity, and 24°C during the day and 22°C at night. For VIGS of *FLS2* in N. benthamiana, Agrobacterium tumefaciens GV3101 carrying pTRV1 or pTRV2-*FLS2* (53) was suspended in induction buffer (10 mM MES, 200 μM acetosyringone, pH 5.5) at OD_600_ = 0.5 and incubated at 28°C under shaking at 200 rpm for 3 h. pTRV2-*PDS* and pTRV2-*EC1* were used as positive and negative controls, respectively (24). The cells were collected by centrifugation at room temperature, 3,000 × *g* for 2 min. *Agrobacterium* cells carrying pTRV1 and pTRV2 with *FLS2*, pTRV2-*PDS*, or pTRV2-*EC1* were mixed at a 1:1 (vol/vol) ratio and resuspended in infiltration buffer (5 mM MES, pH 5.5). The mixtures were inoculated into all leaves of 2-week-old N. benthamiana plants (Fig. S3B). The efficiency of gene silencing was validated by quantitative RT-qPCR analysis. The experiment was repeated at least three times, with multiple replicates.

### RT-qPCR and RT-PCR.

RT-qPCR assays were performed as described previously (54). Peptides at 1 μM were infiltrated into the leaves of N. benthamiana plants. After 3 h, leaf disks (1 cm diameter) were collected for cDNA preparation and RT-qPCR on an ABI QuantStudio6 Flex real-time PCR system (Applied Biosystems, USA). Gene expression levels were standardized to the level of the constitutively expressed *NbEF1α*, normalized to the expression in mock-treated plants, and calculated using the 2^–ΔΔCt^ method.

For RT-PCR, total RNA was extracted using a Bacterial RNA kit (Omega). cDNA was synthesized using the Evo *M-MLV* RT Mix kit with gDNA Clean for qPCR (Accurate Biology). *fliC-1* and *fliC-2* mRNAs were amplified using the primers listed in Table S2. The *recA* gene was employed as a reference. All assays were repeated at least three times.

### Phylogenetic analysis and binding affinity prediction.

The two flagellin-encoding genes were extracted from the genome of *P. kilonensis.* BLAST analysis was used to search for FliC homologs in the P. fluorescens group (taxid: 136843) in NCBI. MEGA X was used for multiple alignments, and the Tamura–Nei model and JTT matrix-based model were used to construct the phylogenetic tree, respectively (55 to 57). Estimates of the binding affinity of Flg22 peptides to FLS2 or BAK1 were calculated using the MutaBind2 server (58). The crystal structure of FLS2-Flg22-BAK1 (PDB: 4MN8) (29) was acquired from the Protein Data Bank (59) and uploaded to the MutaBind2 server. The residues of Flg22 were mutated to Flg22-1 and Flg22-2, respectively, and ΔΔG_bind_ values were obtained (−1.5 ≤ ΔΔG ≤ 1.5 means not deleterious).

### Statistical analysis.

All data were processed using software IBM SPSS Statistics 25.0 (SPSS, Chicago, IL, USA). The data were analyzed using one-way ANOVA, Tukey’s test. *P* <0.05 was considered statistically significant.
