# Supplementary material for: Duplicated Flagellins in Pseudomonas Divergently Contribute to Motility and Plant Immune Elicitation
Source: Microbiol Spectr. 2023 Jan 11;11(1):e03621-22. doi: 10.1128/spectrum.03621-22 (PMC9927476; doi:10.1128/spectrum.03621-22)
Supplement: Supplemental file 2 — Fig. S1 to S8. Download spectrum.03621-22-s0002.pdf, PDF file, 2.2 MB [file spectrum.03621-22-s0002.pdf]

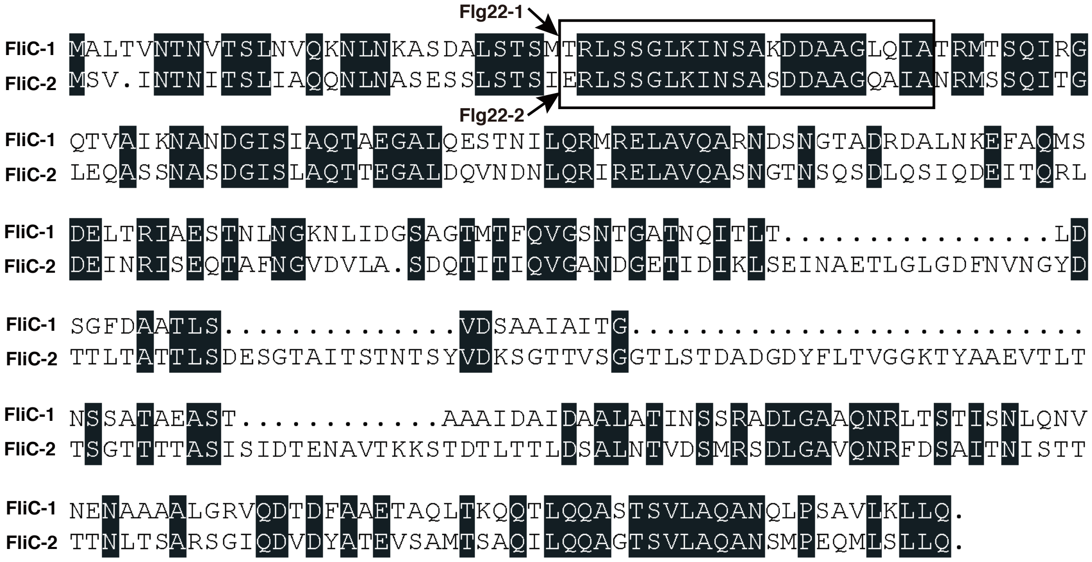

Fig. S1 Alignment of the FliC-1 and FliC-2 amino-acid sequences from *P. kilonensis* F113. Green indicates 100% identity.

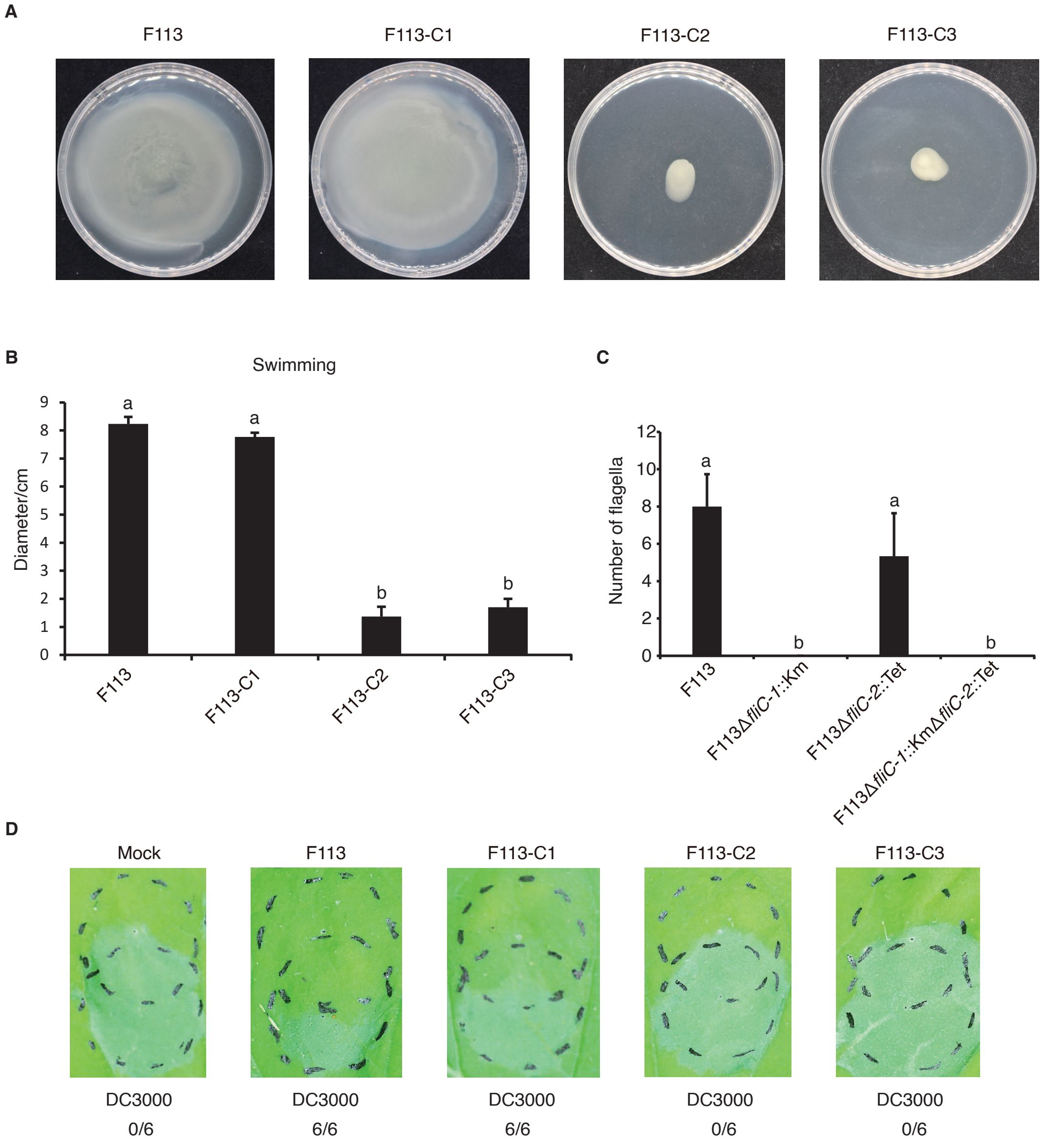

Fig. S2 Analysis of motility of *P. kilonensis* F113 and its derivatives. (A and B) Phenotype and quantitative analysis of swimming motility of *P. kilonensis* F113 and its derivatives. (C) Quantitative analysis of the flagella of *P. kilonensis* F113 and its derivatives. Different letters indicate statistically significant differences between different treatments (one-way ANOVA, Tukey's test; $P < 0.05$). All experiments were repeated three times with similar results. (D) Challenge-inoculation HR assays for functional PTI were conducted by first infiltrating *N. benthamiana* leaves with 1 × 10^8 CFU/mL of the test *Pseudomonas* strains (upper circles). After 6 h, an overlapping inoculation of 5 × 10^6 CFU/mL of the HR-inducing strain *Pst* DC3000 (lower circles) was made. The fraction under each image indicates the number of times that the HR was inhibited compared to the number of test inoculations.

A

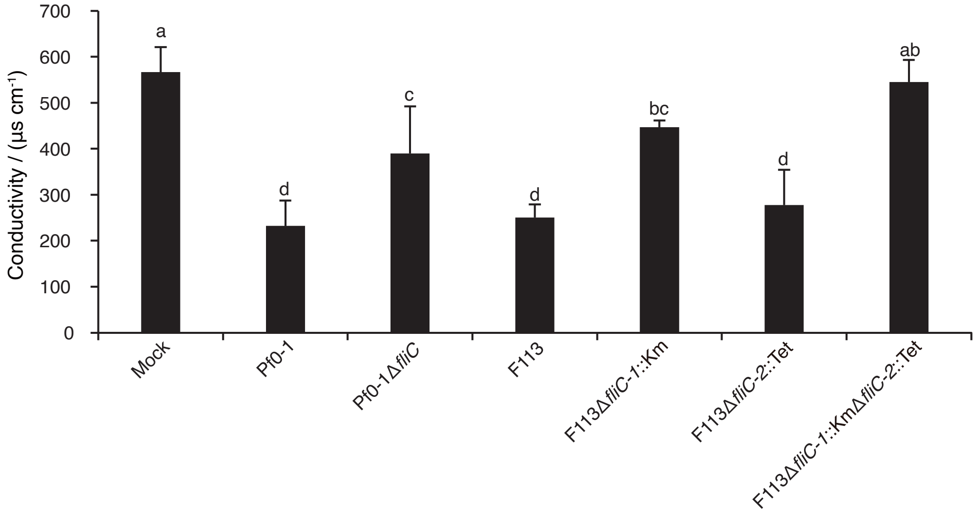

B

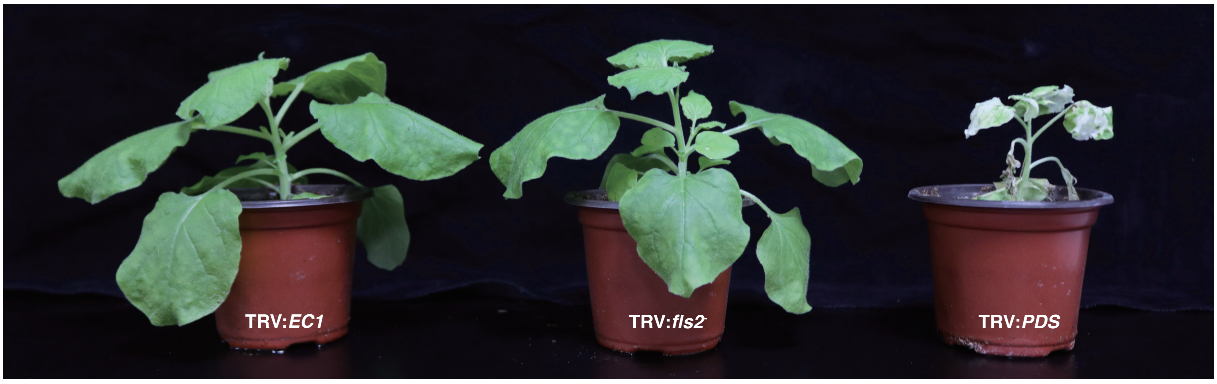

C

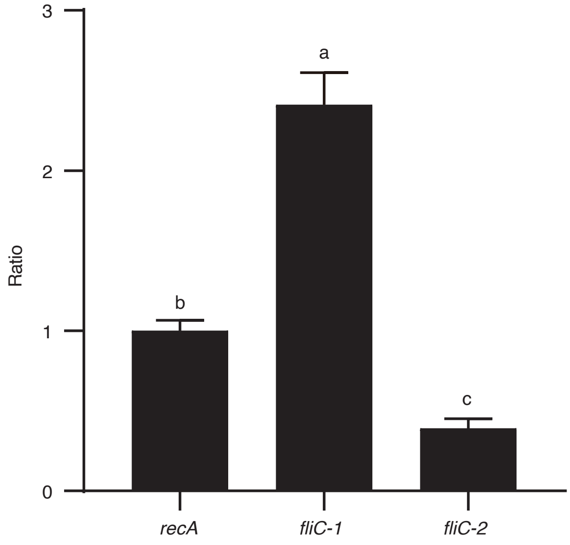

Fig. S3 (A) Ion leakage data of Fig. 3b. (B) Photographs of VIGS plants. Silencing of *PDS* (phytoene desaturase) gene was used to visualize VIGS efficiency. (C) RT-PCR-based quantitative analysis of *fliC-1* and *fliC-2* expression relative to *recA*. Different letters indicate statistically significant differences between different treatments (one-way ANOVA, Tukey's test; $P < 0.05$). All experiments were repeated three times with similar results.

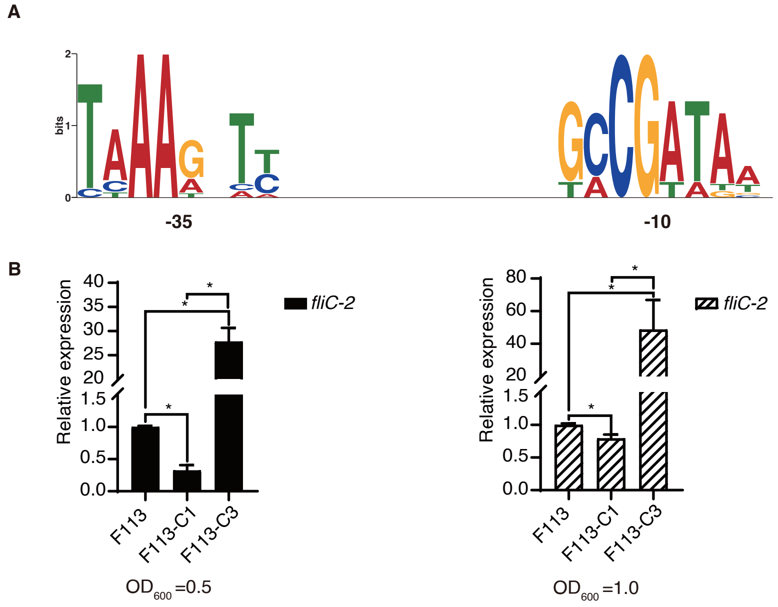

Fig. S4 (A) MEME LOGO showing the motifs of the -35 sites and -10 sites from 13 reported functional flagellin promoters. The height of each letter represents the relative frequency of each base at different positions in the consensus sequence. (B) RT-qPCR analysis of *fliC-1* and *fliC-2* expression in *P. kilonensis* F113 and its derivatives. One-way ANOVA, Tukey's test; *$P < 0.05$.

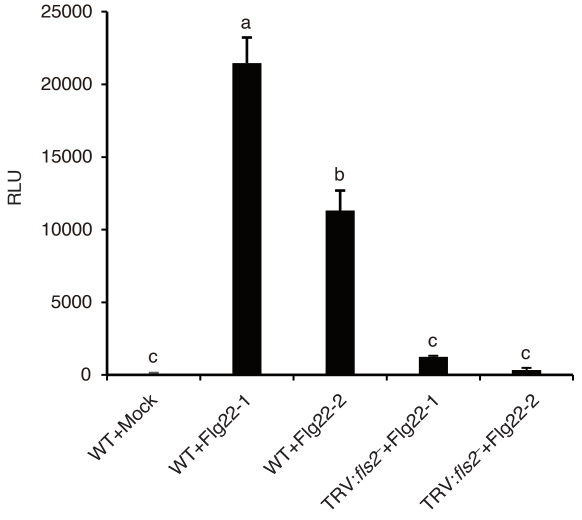

Fig. S5 Total ROS production induced by Flg22 peptides in wild-type and *fls2*-silenced *N. benthamiana* plants. Different letters indicate statistically significant differences between different treatments (one-way ANOVA, Tukey's test; $P < 0.05$). All experiments were repeated three times with similar results.

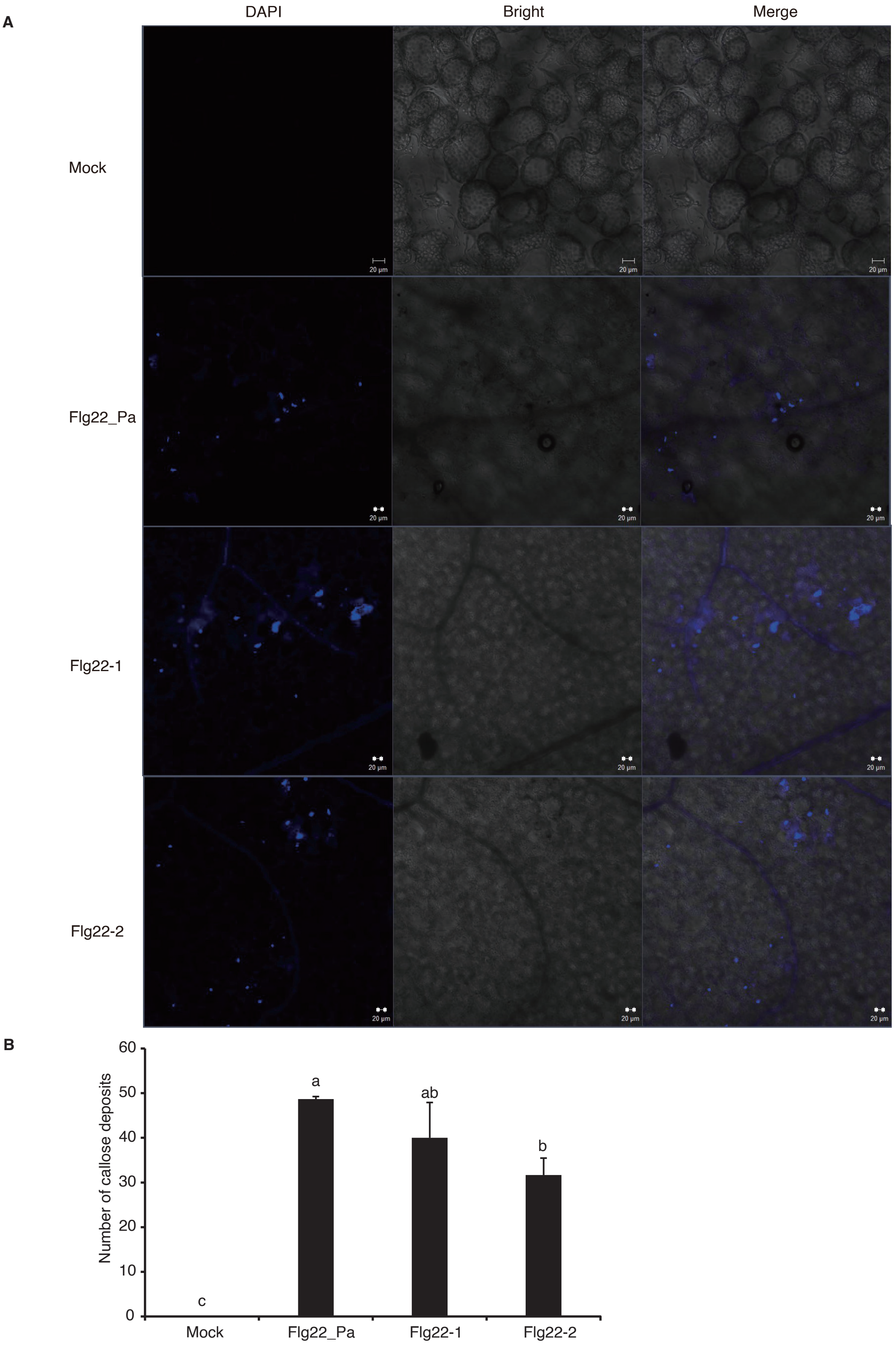

Fig. S6 (A and B) Photographs and quantitative analysis of callose deposition induced by Flg22 peptides in *N. benthamiana*. Different letters indicate statistically significant differences between different treatments (one-way ANOVA, Tukey's test; $P < 0.05$). All experiments were repeated three times with similar results.

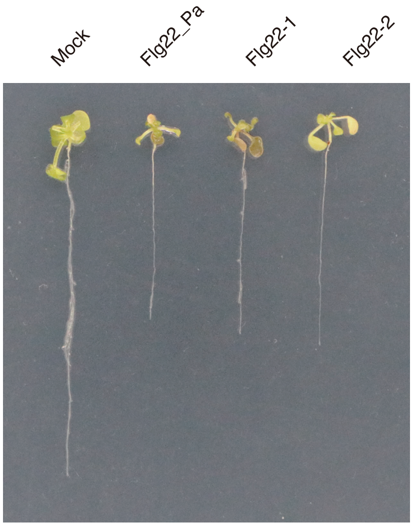

Fig. S7 Effects of various Flg22 peptides on *Arabidopsis* seedling growth. All experiments were repeated three times with similar results.

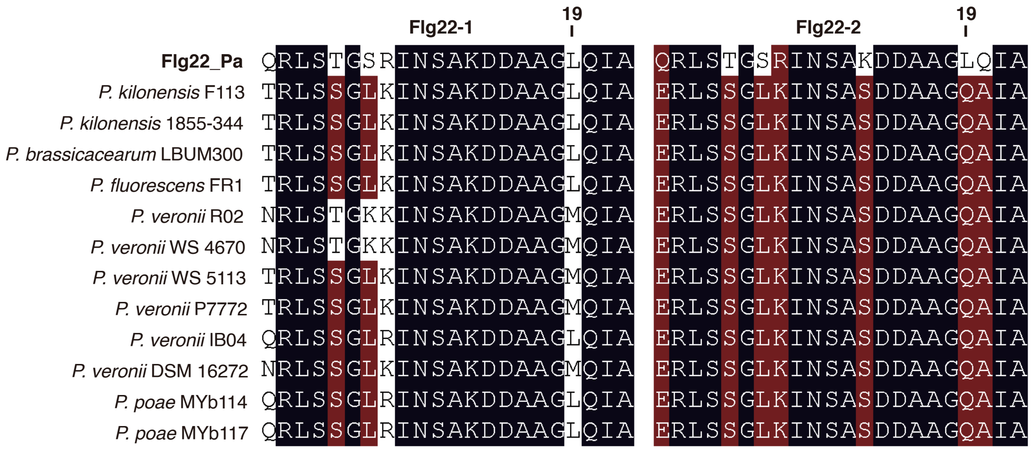

Fig. S8 Sequence alignment of Flg22-1 and Flg22-2 from 12 *Pseudomonas* strains. Dark blue and red indicate 100% and ≥ 75% identity, respectively.

REFERENCE

1. Shanahan P, O'Sullivan D J, Simpson P, Glennon JD, O'Gara F. 1992. Isolation of 2,4-diacetylphloroglucinol from a fluorescent pseudomonad and investigation of physiological parameters influencing its production. Appl Environ Microbiol 58:353-8.
2. Buell CR, Joardar V, Lindeberg M, Selengut J, Paulsen IT, Gwinn ML, Dodson RJ, Deboy RT, Durkin AS, Kolonay JF, Madupu R, Daugherty S, Brinkac L, Beanan MJ, Haft DH, Nelson WC, Davidsen T, Zafar N, Zhou L, Liu J, Yuan Q, Khouri H, Fedorova N, Tran B, Russell D, Berry K, Utterback T, Van Aken SE, Feldblyum TV, D'Ascenzo M, Deng WL, Ramos AR, Alfano JR, Cartinhour S, Chatterjee AK, Delaney TP, Lazarowitz SG, Martin GB, Schneider DJ, Tang X, Bender CL, White O, Fraser CM, Collmer A. 2003. The complete genome sequence of the *Arabidopsis* and tomato pathogen *Pseudomonas syringae* pv. *tomato* DC3000. Proc Natl Acad Sci U S A 100:10181-6.
3. Compeau G, Al-Achi BJ, Platsouka E, Levy SB. 1988. Survival of rifampin-resistant mutants of *Pseudomonas fluorescens* and *Pseudomonas putida* in soil systems. Appl Environ Microbiol 54:2432-8.
4. Wei HL, Chakravarthy S, Worley JN, Collmer A. 2013. Consequences of flagellin export through the type III secretion system of *Pseudomonas syringae* reveal a major difference in the innate immune systems of mammals and the model plant *Nicotiana benthamiana*. Cell Microbiol 15:601-18.
5. Zhao H, Liu YP, Zhang LQ. 2019. In silico and Genetic Analyses of Cyclic Lipopeptide Synthetic Gene Clusters in *Pseudomonas* sp. 11K1. Front Microbiol 10:544.
6. Figurski DH, Helinski DR. 1979. Replication of an origin-containing derivative of plasmid RK2 dependent on a plasmid function provided in trans. Proc Natl Acad Sci U S A 76:1648-52.
7. Kovach ME, Elzer PH, Hill DS, Robertson GT, Farris MA, Roop RM, 2nd, Peterson KM. 1995. Four new derivatives of the broad-host-range cloning vector pBBR1MCS, carrying different antibiotic-resistance cassettes. Gene 166:175-6.
8. Barahona E, Navazo A, Garrido-Sanz D, Muriel C, Martínez-Granero F, Redondo-Nieto M, Martín M, Rivilla R. 2016. *Pseudomonas fluorescens* F113 Can Produce a Second Flagellar Apparatus, Which Is Important for Plant Root Colonization. Front Microbiol 7:1471.
9. Inoue YH, Kutsukake K, Iino T, Yamaguchi S. 1989. Sequence analysis of operator mutants of the phase-1 flagellin-encoding gene, *fliC*, in *Salmonella typhimurium*. Gene 85:221-6.
10. Helmann JD. 1991. Alternative sigma factors and the regulation of flagellar gene expression. Mol Microbiol 5:2875-82.
11. Belas R. 1994. Expression of multiple flagellin-encoding genes of *Proteus mirabilis*. J Bacteriol 176:7169-81.
12. DeShazer D, Brett PJ, Carlyon R, Woods DE. 1997. Mutagenesis of *Burkholderia pseudomallei* with Tn5-OT182: isolation of motility mutants and molecular characterization of the flagellin structural gene. J Bacteriol 179:2116-25.

13. Studholme DJ, Buck M. 2000. The alternative sigma factor sigma(28) of the extreme thermophile *Aquifex aeolicus* restores motility to an *Escherichia coli fliA* mutant. FEMS Microbiol Lett 191:103-7.
14. Lee MC, Weng SF, Tseng YH. 2003. Flagellin gene *fliC* of *Xanthomonas campestris* is upregulated by transcription factor Clp. Biochem Biophys Res Commun 307:647-52.
15. Okuda J, Murayama F, Yamanoi E, Iwamoto E, Matsuoka S, Nishibuchi M, Nakai T. 2007. Base changes in the *fliC* gene of *Edwardsiella tarda*: possible effects on flagellation and motility. Dis Aquat Organ 76:113-21.
16. Poggio S, Aguilar C, Osorio A, González-Pedrajo B, Dreyfus G, Camarena L. 2000. sigma(54) Promoters control expression of genes encoding the hook and basal body complex in *Rhodobacter sphaeroides*. J Bacteriol 182:5787-92.
17. Leal-Morales A, Pulido-Sánchez M, López-Sánchez A, Govantes F. 2022. Transcriptional organization and regulation of the *Pseudomonas putida* flagellar system. Environ Microbiol 24:137-157.
18. Totten PA, Lory S. 1990. Characterization of the type a flagellin gene from *Pseudomonas aeruginosa* PAK. J Bacteriol 172:7188-99.
